# Urinary interleukin-6 as a predictor of radiographic progression in rheumatoid arthritis: A 3-year evaluation

**DOI:** 10.1038/srep35242

**Published:** 2016-10-12

**Authors:** Yune-Jung Park, Seung-Ah Yoo, Ga-Ram Kim, Chul-Soo Cho, Wan-Uk Kim

**Affiliations:** 1 Department of Internal Medicine, Division of Rheumatology, St. Vincent’s Hospital, College of Medicine, The Catholic University of Korea, Suwon, Korea; 2 Center for Integrative Rheumatoid Transcriptomics and Dynamics, The Catholic University of Korea, Seoul, Korea; 3 Department of Internal Medicine, Division of Rheumatology, Yeouido St. Mary’s Hospital, College of Medicine, The Catholic University of Korea, Seoul, Korea; 4 Department of Internal Medicine, Division of Rheumatology, Seoul St. Mary’s Hospital, College of Medicine, The Catholic University of Korea, Seoul, Korea

## Abstract

Previously, we demonstrated that the urine proteome signature of patients with rheumatoid arthritis (RA) reflects inflammation-related cellular processes. Here, we measured interleukin (IL)-6, IL-8, and chemokine ligand 2 (CCL2) concentrations in the urine of RA patients and prospectively investigated their role in predicting RA activity and prognosis. One hundred seventy-three RA patients and 62 non-RA controls were recruited. Urinary IL-6, CCL2, and IL-8 levels were elevated in RA patients and correlated well with disease activity. Urinary IL-6 level at presentation was an independent risk factor of radiographic progression at 1 and 3 years. High urinary IL-6 level increased the risk ratio of radiographic progression by 2.9-fold, which was comparable to high serum CRP. Moreover, combination of urinary IL-6 and serum CRP measures synergistically increased the predictability of radiographic progression. In a subgroup with normal ESR, patients with the highest tertile of urinary IL-6 were at 6.4-fold greater risk of radiographic progression. Conclusively, high urinary IL-6 level at presentation is an independent risk factor for radiographic progression of RA, reflecting disease activity. Urinary IL-6 in combination with serum CRP may be a useful parameter for estimating RA prognosis.

Rheumatoid arthritis (RA) is a systemic inflammatory disease primarily affecting joints^1^. Chronic inflammation of the joints leads to bone and cartilage destruction and deformity^1^,^2^, which progressively manifest as bone erosion, joint space narrowing, and ankylosis of the joints on radiography. Despite numerous studies, there is ongoing controversy as to which biomarkers can adequately predict disease progression in RA patients as a prognostic factor. The blood markers most commonly used to predict rapid progression include the presence of rheumatoid factor (RF), anti-cyclic citrullinated peptide antibody (ACPA), and erythrocyte sedimentation rate (ESR) and C-reactive protein (CRP) levels^3^,^4^,^5^. However, these markers account for only 32% of the total variance in joint destruction prediction^6^. Therefore, there is an unmet need for additional biomarkers that will allow early identification of patients with a potentially aggressive disease course.

For many diseases, urine can be used to provide complementary information to that collected via serum measures^7^,^8^,^9^. Some urinary chemokines including chemokine (C-C motif) ligand 2 (CCL2) from SLE patients can be potential biomarkers for detecting preclinical renal damage and monitoring renal flares^7^. Urinary collagen fragments also may be useful for early diagnosis of diabetic nephropathy^8^. Previously, we performed quantitative proteome profiling of urine samples from RA patients and non-RA controls using liquid chromatography−tandem mass spectrometry (LC−MS/MS) analysis^9^. Through integrative analysis with transcriptomes of synovial tissues, we demonstrated that proteomic signatures in the urine of RA patients reflect inflammation-related cellular processes, including cell adhesion, immune response, and proteolysis. Such results indicate that the urine of RA patients can serve as a source for identifying surrogate biomarkers that represent joint inflammation, which may be associated with the excretion of overproduced proteins, including pro-inflammatory cytokines and chemokines, from chronic inflammatory cells^9^.

Analysis of cytokine mRNA and protein in synovial tissues has revealed that a variety of pro-inflammatory cytokines including tumor necrosis factor (TNF)-α, interleukin (IL)-1, and IL-6, as well as chemokines including CCL2 and IL-8 are upregulated in the peripheral blood and the joints of RA patients^10^. In this study, we postulate that the urine of RA patients reflects systemic inflammatory status and thus urinary cytokine and chemokine levels, including IL-6, IL-8, and CCL2, could be potential biomarkers of disease activity and prognosis of RA. To address this issue, we prospectively investigated the relationship between urinary IL-6, IL-8, and CCL2 levels at initial presentation and radiographic progression of RA, which was monitored over three years.

## Results

### Characteristics of the study population

**Figure 1:**
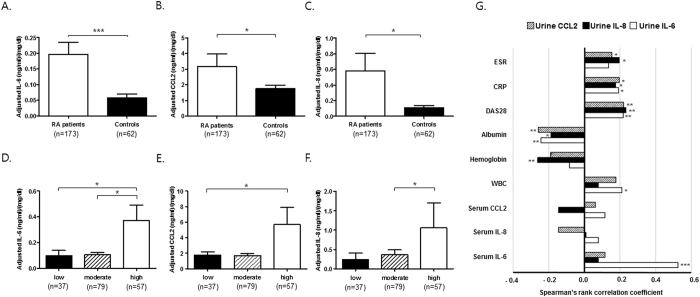
IL-6, CCL2, and IL-8 concentrations in the urine of RA patients and their association with disease activity. (**A**–**C**) Comparison of urinary levels of IL-6 (**A**), CCL2 (**B**), and IL-8 (**C**) adjusted for urine creatinine between RA and non-RA controls. IL-6, CCL2, and IL-8 concentrations were determined in urine samples from 173 RA patients and 62 non-RA controls by ELISA. (**D**–**F**) Adjusted levels of IL-6 (**D**), CCL2 (**E**), and IL-8 (**F**) according to RA disease activity, which was assessed by DAS28; low (DAS28 score <3.2), medium (3.2 ≤ DAS28 score <5.1), and high (DAS28 score ≥5.1). (**G**) Correlation of adjusted urinary IL-6, CCL2, and IL-8 with conventional blood parameters of inflammation in RA patients. The bar graphs show the median and upper interquartile range. (**A**–**F**) Y axis - unit length is (ng/ml)/(mg/dl). (**G**) X axis - unit length is Spearman’s rank correlation coefficient. *P < 0.05. **P < 0.01, ***P < 0.001.

Baseline demographic and disease characteristics are presented in Table 1. Radiographic scores at baseline were 39.5 [interquartile range (IQR): 8.0 to 64.5]. Changes (delta modified Sharp van der Heijde score: ΔSHS > 5) from baseline to 1 year and from baseline to 3 years were 11.0 [IQR: 5.0 to 23.0] (n = 34) and 28.0 [IQR: 15.0 to 66.0] (n = 116), respectively. After adjustment for urine creatinine, urinary levels of the three cytokines (IL-6, CCL2, and IL-8) (n = 173) were significantly higher in RA patients than in non-RA control subjects (n = 62) (Fig. 1A,C), which concurs with our previous report demonstrating that RA patients have inflammatory urine^9^. In RA patients, there were no differences in levels of the three urinary cytokines according to age (≤60 versus >60 years), sex, disease duration (disease duration tertile) or use of medications, including prednisolone, methotrexate, leflunomide, hydroxychloroquine, or anti-TNF-α therapy as assessed by the Mann-Whitney U-test or the Kruskall-Wallis test (data not shown); the non-parametrical Kruskall-Wallis test was used to investigate the differences between more than two subgroups as obtained by categorization according to age or disease duration tertile.

### Correlation of urinary IL-6, CCL2, and IL-8 levels with RA disease activity

When the RA patients were divided into three groups based on 28-joint disease activity score (DAS28) (low: DAS28 ≤ 3.2 [n = 37], moderate: 3.2 < DAS28 ≤ 5.1 [n = 79], and high disease activity: DAS28 > 5.1 [n = 57]), urinary IL-6, CCL2, and IL-8 levels significantly increased in the subgroup with high disease activity (Fig. 1D,F). In univariate analysis, urinary IL-6, CCL2, and IL-8 levels showed positive correlations with serum CRP levels and DAS28 (Fig. 1G), indicating that urinary cytokines and chemokines reflect systemic inflammation in RA patients. In particular, urinary IL-6 levels were most significantly correlated with other parameters of systemic inflammation including serum albumin levels, white blood cell counts, and serum IL-6 concentrations; the correlation coefficient between serum and urine IL-6 levels was 0.526 (*P* < 0.001) (Fig. 1G).

### Urinary IL-6 levels for prediction of rapid radiographic progression at 1 year

**Figure 2:**
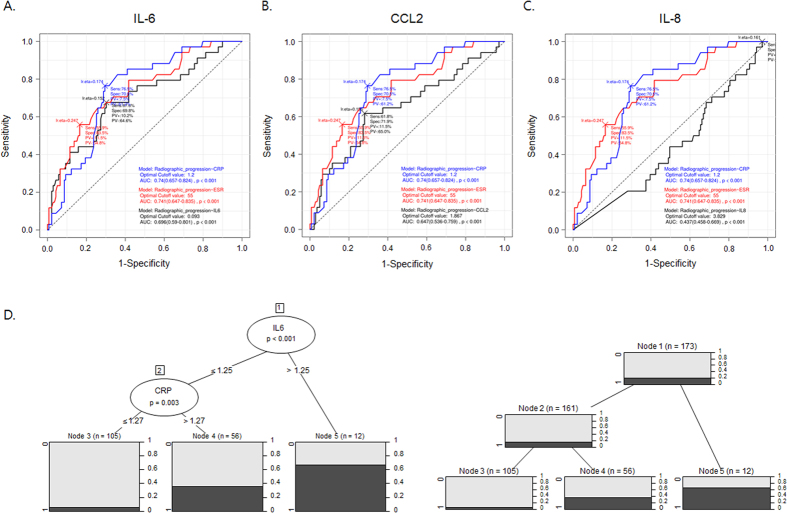
Predictive value of urinary IL-6 for radiographic progression of RA. (**A**–**C**) ROC curve analyses of CRP, ESR, urinary IL-6 (**A**), urinary CCL2 (**B**), and urinary IL-8 (**C**) levels adjusted for urine creatinine for assessing the prediction accuracy of radiographic progression. (**D**) Decision tree for predicting radiographic progression in RA patients. At each node, the best predictor for radiographic progression was selected from multidimensional potential barriers and then the optimal forecasting values were determined. The cut-off values of urinary IL-6 and CRP were 1.25 [(ng/mL)/(mg/dL)]×1000 and 1.27 mg/dl, respectively. The accuracy, sensitivity, 1-specificity, and detection rate of this model were 0.971, 0.765, 0.780, and 0.827, respectively. The dark gray and light blue bars represent patients with radiographic progression and those without, respectively.

The discriminative capacity of urinary IL-6, CCL2, IL-8, CRP, and ESR at baseline for rapid radiographic progression was illustrated by receiver-operating-characteristic (ROC) plots of ΔSHS from baseline to 1 year. The area under the curve (AUC) for adjusted urinary IL-6 was 0.70 [95% CI: 0.59 to 0.80], that for adjusted urinary CCL2 was 0.65 [0.54 to 0.76], and that for adjusted urinary IL-8 was 0.44 [95% CI: 0.46 to 0.67]. Interestingly, the AUC of urinary IL-6 was comparable to that of the CRP (0.74 [95% CI: 0.66 to 0.82]) or ESR (0.74 [95% CI: 0.65 to 0.84]) (Fig. 2A–C). No significant difference in AUC was found between urinary IL-6 levels and CRP or ESR, indicating that the urinary IL-6 concentration at presentation shows fair accuracy for the radiographic progression of RA, and is comparable to CRP and ESR levels in that regard.

We next performed decision tree analysis to classify RA patients using a combination of urinary IL-6, CCL2, and IL-8 and other conventional inflammatory markers. Several decision tree models were automatically generated by the system. The best decision tree model was selected according to the diagnostic sensitivity, specificity, positive predictive value, negative predictive value, detection rate, and accuracy. As shown in Fig. 2D, the selected decision tree consisted of 2 terminal nodes, urinary IL-6 and blood CRP. The respective cut-off values were 1.25 for adjusted urinary IL-6 and 1.27 mg/dL for CRP. The sensitivity, 1-specificity, detection rate, and overall accuracy for diagnosing rapid radiographic progression were 97.1%, 76.5%, 78.0% and 82.7%, respectively.

### Urinary IL-6 levels for prediction of radiographic progression at 3 years

We also investigated the association between urinary IL-6 levels at presentation and 3-year radiographic progression, which was defined as ΔSHS from baseline to 3 years >5. As expected, greater urinary IL-6 levels were associated with a higher risk of radiographic progression at 3 years. In particular, RA patients (n = 57) in the highest tertile (tertile 3, adjusted IL-6 levels ≥116.9) of urinary IL-6 showed a 2.9-fold increase in radiographic progression as compared with those (n = 56) in the lowest tertile (tertile 1, adjusted IL-6 levels <29.9) (Table 2). Serum CRP levels showed a similar risk ratio (odds ratio = 2.8; 95% CI, 1.3 to 6.4, Table 2). In contrast, urinary IL-8 and CCL2 levels at presentation did not significantly increase the risk ratio. The kind of disease modifying anti-rheumatic drugs (DMARDs) or anti-TNF-α therapy did not influence these results (Table 2, Model 2). We further analyzed whether urinary IL-6 plus serum CRP could better predict radiographic progression than IL-6 or serum CRP alone. The results indicated that combination of urinary IL-6 and serum CRP measures synergistically increased the predictability of radiographic progression. (Table 3). Interestingly, patients in the highest urinary IL-6 tertile had 4.5-fold increased risk of radiographic progression without considering CRP (odds ratio = 4.5; 95% CI, 1.3 to 16.4), which was higher than the risk ratio of those in the highest tertile of CRP not considering IL-6 (Table 3).

To determine whether a combination of urinary proteins helps to better predict the risk of radiographic progression, we also stratified our patients according to IL-6, IL-8, and CCL2 tertiles and then compared radiographic severity between the subgroup with two high tertiles (e.g. high IL-6 plus high IL-8, high IL-6 plus high CCL2 or high CCL2 plus high IL-8) and the rest of subgroups. As a result, patients both high in IL-6 and CCL2 or those high in IL-8 and CCL2 showed a higher risk ratio at 3 years than those with high in each single parameter (Supplementary Table 1), indicating that combinatory determinations of urinary IL-6, IL-8, and CCL2 can increase the predictability for radiographic progression.

Univariate analysis showed significant associations between radiographic progression at 3 years and the presence of ACPA, urinary IL-6, CRP, and ESR (data not shown). In stratified analyses in the ACPA-positive patients, the risk ratio for radiographic progression at 3 years was 5.0-fold greater in the both high subgroup (adjusted IL-6 levels ≥116.9 and CRP level ≥1.5 mg/dL) than in the both low subgroup (adjusted IL-6 levels <29.9, CRP level <0.2 mg/dL) (Table 3). Analysis of the subgroup with high ESR revealed that the risk ratio for radiographic progression at 3 years was 6.7-fold higher in the both high subgroup (Table 3). Interestingly, in the subgroup with normal ESR, patients with the highest tertile of urinary IL-6 showed a 6.4-fold increase in radiographic progression at 3 years, although they did not have high levels of serum CRP at presentation. These data suggest that urinary IL-6 can serve as a complementary biomarker for predicting RA prognosis even in those with normal ESR.

## Discussion

Low molecular weight molecules including metabolic products can be found in the urine, because urine is more stable than other bio-fluids^11^. Urine sampling is noninvasive, requires no pre-sampling preparation, and substantially improves compliance. Thus, it may serve as an ideal sample source of biomarkers^12^. Research on the use of urinary biomarkers to facilitate diagnosis and prognosis of diseases outside the urogenital tract, including asthma, colon cancer, and inflammatory bowel diseases, has become increasingly popular^13^,^14^,^15^,^16^. However, few studies have investigated the association of urinary proteins with systemic inflammatory status, and especially with RA prognosis. In this prospective study, we first identified that urine levels of IL-6, IL-8, and CCL2, representative pro-inflammatory cytokines and chemokines, were elevated in RA patients, supporting the view that the urine of RA patients is pro-inflammatory in nature and reflects systemic inflammation^9^. In particular, a high urinary IL-6 level was independently associated with the radiographic progression of RA, suggesting that IL-6 is comparable to current tests when it used alone in the prediction of RA prognoses.

Previously, we introduced a novel approach for assessing RA activity through urinary proteome profiling^9^, and found that 296 differentially expressed proteins identified in the urine of RA patients were involved in inflammation-related cellular process^9^. IL-6 is one of the most abundantly expressed cytokines identified in the sera of RA patients^10^. It promotes B cell growth and differentiation, Th17 cell generation, and osteoclast formation^17^,^18^,^19^,^20^,^21^. Since the molecular weight of IL-6 is about 26 kDa^22^, it can be easily detected in urine. In the current study, multivariate logistic regression analysis revealed that urinary IL-6 at presentation is an independent predictor for radiographic progression of RA at 1 and 3 years. Higher IL-6 levels reflect cellular damage, such as oxidative stress^23^ and are involved in the regulation of bone metabolism^21^. Our data suggest that urinary IL-6 is a useful and non-invasive indicator of radiographic progression of RA.

Chemokines can be divided into 2 major subfamilies, 1) CXC chemokine including IL-8 and 2) CC chemokines including CCL2^24^,^25^,^26^. Here, we investigated the clinical significance of elevated IL-6, IL-8, and CCL2 concentrations in the urine of RA patients, focusing on their role in predicting RA activity and prognosis. The current study was the first to demonstrate that urinary IL-6, IL-8, and CCL2 levels were positively correlated with inflammatory indices of RA, including serum CRP and DAS28 levels. Previous studies have shown that CCL2 levels are elevated in the blood and synovial fluids of RA patients and are correlated with RA disease activity^27^,^28^,^29^,^30^. Injection of CCL2 antagonist markedly reduces the severity of experimentally-induced arthritis^31^. The role of IL-8 in the migration of inflammatory cells has been highlighted in studies of neutrophil trafficking^32^. In RA, IL-8 stimulates osteoclast activation and its level reflects joint damage^33^,^34^,^35^. Our results, together with previous reports^32^,^33^,^34^,^35^,^36^,^37^,^38^, indicate that IL-6, IL-8, and CCL2 levels in urine reflect RA disease activity and therefore may be useful for monitoring disease activity in RA patients.

It is unclear whether increased urinary IL-6 levels originate from inflamed synovia or urogenital tracts. RA patients have a high prevalence of subclinical nephropathy, exhibiting microalbuminuria and tubular dysfunction^36^,^37^, which can affect the excretion of urinary proteins including IL-6. In the current study, we found that glomerular filtration rate (GFR), which was estimated using the renal disease equation, did not vary between RA and osteoarthritis (Table 1). However, proteinuria levels adjusted for urine creatinine were significantly increased in the urine of RA patients compared to non-RA control subjects (Table 1). Moreover, urinary IL-6 levels were positively correlated with the degree of proteinuria (r = 0.481, *P* < 0.001, data not shown), whereas serum IL-6 levels were not (r = 0.033, *P* = 0.734). Moreover, RA patients with radiographic progression showed elevated urinary IL-6 levels in all the subgroups divided by proteinuria tertile (Supplementary Figure 1A,B). In contrast, the probability plot indicated that higher serum CRP and ESR further increased the probability of radiographic progression with elevated urinary IL-6 levels in patients with RA (Supplementary Figure 1C,D). These data suggest that 1) subclinical renal damage is frequently observed in RA patients; 2) elevated IL-6 in the urine of RA patients may be associated with subclinical nephropathy; and 3) proteinuria itself does not influence RA disease severity, unlike urinary IL-6, ESR and serum CRP.

Another possibility is that elevated IL-6 levels in RA urine can be a consequence of systemic inflammatory response in RA patients. The protein level in urine often reflects serum protein level^38^. Increased tissue and serum levels of IL-6 have been implicated in the pathology of RA^10^,^22^. In this study, we found that urinary IL-6 levels correlated positively with serum IL-6 levels and other serum inflammatory markers, including ESR, serum CRP, DAS28, and white blood cell levels, while correlating negatively with serum albumin and hemoglobin concentrations (Fig. 1G). These observations, together with previous reports^10^,^17^,^18^,^19^,^20^,^22^,^38^, indicate that the increase in urinary IL-6 level may result from an overflow of circulatory IL-6 under systemic inflammatory conditions, although direct evidence for this is lacking at this stage. Further studies will be required to clarify this issue.

Given the complexity of the immunological networks in RA pathogenesis^1^,^39^,^40^, a single aspect of disease severity may not be sufficient to predict radiographic progression in RA. Although disease activity at presentation, baseline erosion, smoking status, and the presence of autoantibody have been associated with the risk of radiographic progression^3^,^4^,^5^, they may have limited predictive power on an individual basis^6^. The combination of multiple indices can potentially enhance predictive power and may be a more promising approach. IL-6 plays a major role in the synthesis of CRP and other acute phase reactants produced by hepatocytes^41^,^42^,^43^,^44^. Since IL-6 is an upstream regulator for the production of CRP and other acute phage reactants, it can provide additional benefits over current serum markers, including CRP (Table 3).

In this study, we found that a panel of high IL-6 plus high CCL2 or that of IL-8 plus CCL2 could better predict radiographic progression at 3 years than each single parameter. Additionally, RA patients with high IL-6 and CRP levels (both high) had a greater risk ratio for radiographic progression at 1 and 3 years than IL-6 or serum CRP alone (Table 3). In particular, in the subgroup with normal ESR, patients with the highest tertile of urinary IL-6 showed a 6.4-fold increase in radiographic progression at 3 years, despite low and moderately high levels of serum CRP at presentation. Taken together, these results suggest that urinary IL-6 is complementary to conventional measures of predicting RA prognosis.

The current study has certain limitations. First, this study was conducted on an Asian population, so it is not clear whether RA patients from other ethnicities will have the same results. Second, we did not take into consideration the influence of many factors, including menstrual variations, race, medication, and diet, on urinary creatinine levels^45^. While avoiding the collection of urine samples during menstruation or vaginal discharge, we did not ask the menstrual status of every patient, which is another limitation of the current study. Finally, given the diurnal variation of serum cytokine levels^46^, it remains unclear if static cytokine concentrations in spot urine samples, which were collected in the morning between 8 and 10 AM, stably represent cytokine dynamics for 24 hours. Further studies will be required to clarify the aforementioned compounding factors.

In summary, in a single-center prospective study, we found that urinary IL-6, IL-8 and, CCL2 levels were elevated in RA patients and correlated well with disease activity. In particular, urinary IL-6 concentration at presentation helps to predict radiographic progression of RA at 1 and 3 years. Moreover, the combination of urinary IL-6 and serum CRP synergistically increased the predictability of radiographic progression. These results suggest that urinary IL-6 in combination with serum CRP may increase the specificity of ongoing inflammation assessment and could be promising parameters for predicting radiographic progression of RA.

## Methods

### Ethics Statement

The study protocol was approved by the Institutional Review Board of the Catholic Medical Center (VC12RISI0191). All protocols were performed in accordance with relevant guidelines and regulations. Written informed consent was obtained from all human participants after complete description of the study.

### Study population

One hundred seventy-three patients with RA and 62 non-RA control subjects were consecutively recruited from outpatients of Seoul St. Mary’s Hospital and St. Vincent’s Hospital in Korea. All RA patients fulfilled the 1987 American College of Rheumatology criteria for RA^47^. Non-RA controls were recruited from subjects without inflammatory arthritis, including 15 healthy subjects, 11 patients with fibromyalgia, and 26 with osteoarthritis. The following subjects were excluded: those with severe cardiac diseases, renal diseases that could affect GFR, current or chronic infections, pregnancy, or a history of malignancy.

### Disease activity and radiographic outcomes

RA disease activity was evaluated using DAS28 based on ESR at 3-month follow-up^48^. Disease severity of RA was assessed by evaluating radiographic damage on X-rays of the hands and feet of subjects. Radiographs of the hands and feet were taken at baseline and annually thereafter. Radiographic severity was scored by two rheumatologists blinded to study protocol in chronologic order for erosions and joint space narrowing according to the SHS method^49^. Patients whose SHS increased by more than five points (ΔSHS > 5) from baseline to 1 year were considered to have rapid radiographic progression^50^,^51^,^52^.

### Enzyme-linked immune sorbent assay (ELISA)

First or second morning urine samples were collected in sterile 500 mL plastic tubes containing 0.05% sodium azide. Urine samples were centrifuged at 14,000 × *g* for 10 minutes at 4 °C immediately after collection, and the clarified supernatants were aliquoted into sterile 1 mL tubes and stored at −70 °C until used. Urinary concentrations of IL-6, CCL2, and IL-8 were determined by ELISA, as described previously^9^.

### Statistical Analysis

Comparisons between RA patients and control groups were made using the Mann-Whitney U-test for continuous variables and chi-square statistics for categorical variables. When IL-6, IL-8, CCL2, and CRP levels were used as continuous variables, values were log transformed to normalize the distributions. Spearman correlation coefficients were used to describe the association between variables. To evaluate the ability of urinary cytokines to predict rapid radiographic progression (ΔSHS > 5 after 1 year), ROC curves were plotted. Decision tree analyses were used to divide the data set into subsets with the best discrimination between radiographic progression and non-progression groups. The cut-off value of the variable for patient regrouping was obtained from the decision tree program using R version 3.2.3 software. The best decision tree model was identified based on the sensitivity and specificity of the prediction. IL-6, IL-8, CCL2, and CRP tertiles derived from the cohort sample were used to determine associations with 3-year radiographic progression (ΔSHS > 5 after 3 years). To test the hypothesis that the combined measure of IL-6 and CRP levels may synergistically increase the risk ratio of radiographic progression, we created five mutually-exclusive groups based on the combined IL-6 and CRP measure. IL-6 levels were adjusted for urine creatinine and expressed as a conversion value, which was calculated as: IL-6 concentration (ng/mL)/creatinine (mg/dL)×1000. The high extreme group (both high) included those with IL-6 and CRP levels in the highest tertile (adjusted IL-6 levels ≥116.9 and CRP level ≥1.5 mg/dL). The low extreme (both low), which was considered the reference category in this analysis, included only participants with values in the lowest tertile (adjusted IL-6 levels <29.9, CRP level <0.2 mg/dL). The three intermediate categories were defined as an IL-6 level in tertile 3 and a CRP level less than tertile 3 (high IL-6), a CRP level in tertile 3 and an IL-6 level less than tertile 3 (high CRP), and either a CRP or IL-6 level in tertile 2, but with neither in the upper tertiles (both mid). The high ESR group was defined as an ESR level ≥(age/2) mm/hour for men and ≥(age + 10/2) mm/hour for women. Multivariable logistic regression analysis was used to determine the contribution of each variable to radiographic progression. Multivariate models included all covariates with associations from univariate models with a *P*-value ≤ 0.20. All reported *P-*values were two-tailed with a *P*-value of 0.05 indicating statistical significance. Data analyses were performed using R version 3.2.3 (http://www.R-project.org and web-r.org) software.

## Additional Information

**How to cite this article**: Park, Y.-J. *et al*. Urinary interleukin-6 as a predictor of radiographic progression in rheumatoid arthritis: A 3-year evaluation. *Sci. Rep.* **6**, 35242; doi: 10.1038/srep35242 (2016).

## Supplementary Material

Supplementary Information

## Figures and Tables

**Table 1:** Characteristics of participants*.

Characteristics	Non-RA controls (n = 62)	RA (n = 173)	*P*-value†
Age, years	55.0 (49.0–57.0)	53.5 (46.0–61.0)	0.948
Duration of disease, year	1.0 (0.0–4.0)	4.0 (2.0–12.5)	<0.001
Female, n (%)	58 (93.5)	151 (87.2)	0.239‡
Current smoker, n (%)	7 (11.3)	24 (13.9)	0.669‡
Body mass index, kg/m^2^	22.9 (21.8–24.4)	22.3 (20.5–25.0)	0.175
Glomerular filtration rate, ml/min/1.73 m^2^	92.0 (80.4–109.1)	97.0 (81.9–112.0)	0.075
Creatinine, mg/dL	0.7 (0.6–0.9)	0.7 (0.6–0.8)	0.543
Proteinuria, mg/day	12.7 (0.0–30.5)	27.4 (0.1–64.5)	0.018
Albumin, mg/dL	4.7 (4.5–4.9)	4.2 (3.9–4.4)	<0.001
Hemoglobin, g/dL	13.0 (12.6–13.7)	12.0 (11.8–13.5)	<0.001
Erythrocyte sedimentation rate, mm/hour	17.0 (9.0–22.5)	33.0 (19.0–55.0)	<0.001
C-reactive protein, mg/dL	0.1 (0.0–0.1)	0.6 (0.1–2.0)	<0.001
Rheumatoid factor, n (%)§	NA	117 (67.6)	NA
ACPA, n (%)§	NA	121 (70.0)	NA
Disease activity score in 28 joints	NA	4.0 (3.0–5.2)	NA
Sharp van der Heijde score	NA	39.5 (8.0–64.5)	NA
Erosion score	NA	14 (5.6–24.1)	NA
Joint space narrowing score	NA	12 (7.0–22.5)	NA
Prednisolone, n (%)	6 (9.7)	130 (75.1)	<0.001‡
Methotrexate, n (%)	NA	117 (67.0)	NA
Hydroxychloroquine, n (%)	NA	119 (68.8)	NA
Sulfasalazine, n (%)	NA	54 (31.2)	NA
Leflunomide, n (%)	NA	103 (59.5)	NA
Anti-TNF-α, n (%)	NA	14 (8.1)	NA
NSAIDs, n (%)	41 (66.1)	106 (61.2)	0.497

NA, not applicable; ACPA, anti-cyclic citrullinated peptide antibody; TNF-α, tumor necrosis factor-α;

NSAIDs, non-steroidal anti-inflammatory drugs.

^*^Data are presented as median (interquartile range) or percentage.

^†^Unless otherwise noted, the Mann-Whitney U test was used.

^‡^The Chi-square test was used.

^§^Indicates antibody positivity. Positive cut-off values were ≥15 IU/ml for RF and >5 U/mL for ACPA.

**Table 2:** Association of urinary IL-6, CCL2, IL-8 and serum C-reactive protein levels with radiographic progression at 3 years.

Inflammatory marker	N	Radiographic progression		
		Progression no. (%)	Odds ratio (95% confidence interval)	
			Model 1*	Model 2†
Interleukin-6				
tertile 1	56	33 (58.9)	1	1
tertile 2	60	37 (61.7)	1.1 (0.5–2.4)	1.7 (0.7–4.2)
tertile 3	57	46 (80.7)	2.9 (1.3–6.8)	2.6 (1.0–6.9)
Interleukin-8				
tertile 1	57	38 (66.7)	1	1
tertile 2	58	36 (62.1)	0.8 (0.4–1.8)	0.7 (0.3–1.8)
tertile 3	58	42 (72.4)	1.3 (0.6–2.9)	1.1 (0.4–2.7)
Chemokine ligand-2				
tertile 1	55	35 (63.6)	1	1
tertile 2	59	40 (67.8)	1.2 (0.5–2.6)	1.2 (0.5–3.0)
tertile 3	59	41 (69.5)	1.3 (0.6–2.8)	1.1 (0.8–1.7)
C-reactive protein				
tertile 1	56	32 (57.1)	1	1
tertile 2	55	35 (63.6)	1.3 (0.6–2.8)	1.1 (0.5–2.7)
tertile 3	62	49 (79.0)	2.8 (1.3–6.4)	3.1 (1.1–8.3)

^*^Model 1 was adjusted for age, sex, smoking status, disease duration, and the presence of anti-cyclic citrullinated antibody (ACPA) positivity.

^†^Model 2 was adjusted for the variables listed in Model 1 plus disease activity score in 28 joints, use of methotrexate, and use of anti-TNF-α. The positive cut-off value for ACPA was >5 U/mL.

**Table 3:** Association of combined urine IL-6 and serum CRP measures with radiographic progression of RA at 3 years.

Categories based on IL-6 and C-reactive protein*	Odds ratio† (95% confidence interval)	Stratified by ACPA‡ Odds ratio† (95% confidence interval)		Stratified by ESR§ Odds ratio† (95% confidence interval)	
		Positive n = 121	Negative n = 52	Elevated n = 102	Normal n = 71
Both low (n = 23)	1	1	1	1	1
Both mid (n = 62)	1.4 (0.5–3.7)	1.9 (0.6–5.9)	1.1 (0.1–8.9)	0.9 (0.2–3.7)	2.0 (0.5–7.7)
High C-reactive protein (n = 31)	3.7 (1.2–12.1)	2.5 (1.9–8.6)	2.3 (0.2–18.2)	3.2 (0.6–16.7)	2.7 (0.4–16.4)
High interleukin-6 (n = 26)	4.5 (1.3–16.4)	4.5 (1.1–18.7)	4.5 (0.3–32.6)	2.9 (0.5–17.2)	6.4 (1.0–33.2)
Both high (n = 31)	4.6 (1.4–15.2)	5.0 (1.2–20.6)	7.5 (0.5–22.7)	6.7 (1.0–25.8)	2.0 (0.4–11.2)

ACNPA, anti-cyclic citrullinated antibody; ESR, erythrocyte sedimentation rate.

^*^Both low (reference): IL-6 level below the first tertile (<29.9) and C-reactive protein level below the first tertile (<0.2 mg/dL); both mid: mid-range values of C-reactive protein or IL-6, but neither high; high C-reactive protein: high C-reactive protein level only (C-reactive protein >1.5 mg/dL); high IL-6: high IL-6 level only (IL-6 > 116.9); both high: both IL-6 and C-reactive protein levels in the highest tertile (IL-6 ≥ 116.9 and C-reactive protein ≥1.5 mg/dL). IL-6 levels were adjusted for urine creatinine and expressed as units [(ng/mL)/(mg/dL)]×1000.

^†^Adjusted for age, sex, smoking status, disease duration, disease activity scores in 28 joints, use of methotrexate, and use of anti-tumor necrosis factor therapy.

^‡^The positive cut-off value for ACPA was >5.0 U/mL.

^§^The positive cut-off values were as follows: ESR for men >(age/2) mm/hour and ESR for women was >[(age + 10)/2] mm/hour.
