# Supplementary material for: Urinary interleukin-6 as a predictor of radiographic progression in rheumatoid arthritis: A 3-year evaluation
Source: Sci Rep. 2016 Oct 12;6:35242. doi: 10.1038/srep35242 (PMC5059680; doi:10.1038/srep35242)
Supplement: Supplementary Information [file srep35242-s1.pdf]

Supplementary Information

Urinary interleukin-6 as a predictor of radiographic progression in rheumatoid arthritis: A 3-year evaluation

Yune-Jung Park[1,2], Seung-Ah Yoo[2], Ga-Ram Kim[2], Chul-Soo Cho[2,3], Wan-Uk Kim[2,4]

[1]Department of Internal Medicine, Division of Rheumatology, St. Vincent's Hospital, College of Medicine, The Catholic University of Korea, Suwon, Korea; [2]Center for Integrative Rheumatoid Transcriptomics and Dynamics, The Catholic University of Korea, Seoul, Korea; [3]Department of Internal Medicine, Division of Rheumatology, Yeouido St. Mary's Hospital, College of Medicine, The Catholic University of Korea, Seoul, Korea; [4]Department of Internal Medicine, Division of Rheumatology, Seoul St. Mary's Hospital, College of Medicine, The Catholic University of Korea, Seoul, Korea.

Supplementary Table 1. Increase in the risk of radiographic progression at 3 years by combination of urinary IL-6, IL-8, and CCL2 tertiles

Categories based on urinary proteins*	Odds ratio† (95% confidence interval)
Categories based on IL-6 and IL-8 levels*	
Both low (n=27)	1
Both mid (n=64)	1.9 (0.6-4.7)
High IL-6 (n=32)	2.7 (0.9-8.1)
High IL-8 (n=26)	3.5 (0.7-11.2)
Both high (n=24)	3.1 (0.9-9.2)
Categories based on IL-6 and CCL2 levels*	
Both low (n=23)	1
Both mid (n=59)	2.9 (0.7-11.9)
High IL-6 (n=31)	3.3 (0.8-13.1)
High CCL2 (n=34)	1.4 (0.1-2.3)
Both high (n=26)	6.2 (1.4-26.4)
Categories based on IL-8 and CCL2 levels*	
Both low (n=25)	1
Both mid (n=56)	2.9 (0.9-14.3)
High IL-8 (n=34)	2.4 (0.2-24.5)
High CCL2 (n=33)	5.0 (0.6-41.4)
Both high (n=25)	6.1 (1.8-37.9)

* Both low (reference): IL-6 level below the first tertile (< 29.9), IL-8 level below the first tertile (< 10.4), and CCL2 level below the first tertile (85.1); both mid: mid-range values of IL-6, IL-8 or CCL2, but neither high; high IL-6: high IL-6 level only (IL-6 > 116.9); high IL-8: high IL-8 level only (IL-8 > 92.0); high CCL2: high CCL2 level only (CCL2 > 195); both high: both IL-6 and IL-8 levels in the highest tertile (IL-6 ≥116.9 and IL-8 ≥ 92.0); both IL-6 and CCL2 levels in the highest tertile (IL-6 ≥116.9 and CCL2 ≥195); both IL-8 and CCL2 levels in the highest tertile (IL-8 ≥92.0 and CCL2 ≥195). IL-6, IL-8, and CCL2 levels were adjusted for urine creatinine and expressed as units [(ng/mL)/(mg/dL)]X1000.

† Adjusted for age, sex, smoking status, disease duration, disease activity scores in 28 joints, use of methotrexate, and use of anti-tumor necrosis factor therapy.

Supplementary Figure Legend

Supplementary Figure 1. Relationship between urine IL-6 level and proteinuria, ESR, and serum CRP for predicting radiographic progression of RA at 3 years. A. (Left) Comparison of proteinuria levels between radiographic progression and non-progression groups at 1 year. (Right) Urinary IL-6 levels between radiographic progression and non-progression groups at 1 year according to the extent of proteinuria (tertiles 1, 2, and 3). **B.** (Left) Comparison of proteinuria levels between radiographic progression and non-progression group at 3 years. (Right) Urinary IL-6 levels between radiographic progression and non-progression groups at 3 years according to the extent of proteinuria (tertiles 1, 2, and 3). **C** and **D**. Predicted probability plot of high urinary IL-6 (≥1.17 ng/ml: upper tertile of urinary IL-6) for radiographic progression in association with serum CRP (**C**) and ESR levels (**D**) in patients with RA (red circles indicate the upper tertile of ESR or CRP: ESR≥45 mm/hour and CRP≥1.34 mg/dl, black circles indicate the lower tertile of ESR or CRP: ESR<45mm/hour and CRP<1.34 mg/dl). *P <0.05 and **P<0.01.

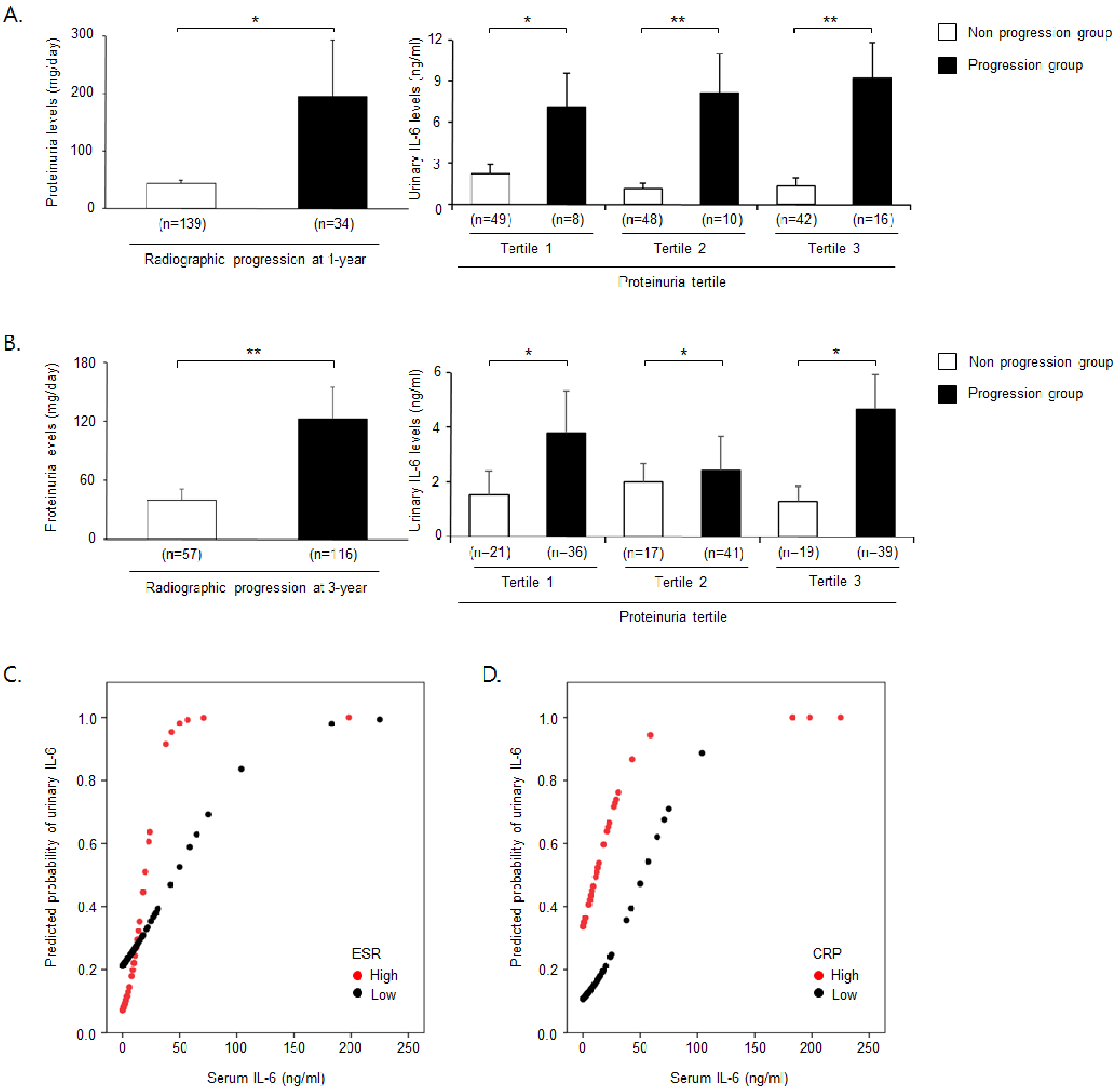
A.
Proteinuria levels (mg/day)
300
200
100
0
*
(n=139)
(n=34)
Radiographic progression at 1-year
Urinary IL-6 levels (ng/ml)
12
9
6
3
0
*
**
**
(n=49)
(n=8)
(n=48)
(n=10)
(n=42)
(n=16)
Tertile 1
Tertile 2
Tertile 3
Proteinuria tertile
Non progression group
Progression group
B.
Proteinuria levels (mg/day)
180
120
60
0
**
(n=57)
(n=116)
Radiographic progression at 3-year
Urinary IL-6 levels (ng/ml)
6
4
2
0
*
*
*
(n=21)
(n=36)
(n=17)
(n=41)
(n=19)
(n=39)
Tertile 1
Tertile 2
Tertile 3
Proteinuria tertile
Non progression group
Progression group
C.
Predicted probability of urinary IL-6
1.0
0.8
0.6
0.4
0.2
0.0
0
50
100
150
200
250
Serum IL-6 (ng/ml)
ESR
High
Low
D.
Predicted probability of urinary IL-6
1.0
0.8
0.6
0.4
0.2
0.0
0
50
100
150
200
250
Serum IL-6 (ng/ml)
CRP
High
Low
